# Lead poisoning in a 6-month-old infant: a case report

**DOI:** 10.3389/fpubh.2023.1132199

**Published:** 2023-05-04

**Authors:** Yifei Duan, Lingyi Yan, Zhengxiang Gao, Yu Gou

**Affiliations:** ^1^Department of Laboratory Medicine, West China Second University Hospital, Sichuan University, Chengdu, Sichuan, China; ^2^Key Laboratory of Birth Defects and Related Diseases of Women and Children, Sichuan University, Ministry of Education, Chengdu, Sichuan, China

**Keywords:** lead, lead poisoning, blood lead level, traditional Chinese medicine, Hu Wang Fen

## Abstract

**Background:**

Lead is a toxic element of the environment that leads to major complications once it enters the blood stream, affecting multiple organs and systems of the body.

**Methods:**

We present a case of a 6-month-old female infant diagnosed with lead poisoning after presenting for routine child health care. The child's mother denied that the infant had a history of exposure to lead-containing substances. After a month of calcium supplementation, the patient's blood lead level remained elevated. We then tested the blood lead level of the mother and father. The results showed that the blood lead level of the mother was 77.0 μg/L and that of the father was 36.9 μg/L. The high blood lead level of the mother attracted our attention. We found that the mother had been using an external traditional Chinese medicine, Hu Wang Fen, which contains lead. After the mother's discontinuation of use of the traditional medicine, the child was treated with symptomatic treatment and chelation therapy. Subsequently, the patient's blood lead level decreased significantly.

**Results:**

Lead toxicity can be a life-threatening problem because of its potential for severe complications. In children, there is no safe blood lead level, and the toxic effects of lead can be prevented by the awareness and avoidance of traditional Chinese medicines that may contain lead.

**Conclusion:**

Even though the diagnosis of lead poisoning remains difficult in children, it must be taken into consideration by the clinician when treating a child using traditional Chinese medicines.

## Introduction

The problem of lead poisoning in children has existed for a long time, and sporadic cases of lead poisoning are reported every year. It is generally believed that the exposure source of lead is most commonly from industry (1) or occupational pollution (2). However, lead poisoning from other sources should not be ignored. Lead poisoning in children is an important health problem; it can lead to growth retardation, intellectual disability and irreversible damage to the nervous system (3), blood system, digestive system and other areas (4), accounting for nearly half of the 2 million lives lost to known chemical exposure in 2019 according to the World Health Organization (5). According to the National Health and Nutrition Examination Survey data, from 2007 to 2010, ~535,000 children aged 1–5 years, or 2.6% of the population, have blood lead levels above 5 μg/dL. More recently, lead exposure has ascribed mainly to household lead contamination. In addition, cases of lead poisoning caused by the use of traditional Chinese medicine have been reported. We report a case of infant lead poisoning due to the mother's use of traditional Chinese medicine. The aim of our work is to sensitize practitioners and parents to the abuse of traditional Chinese medicine in general and draw their attention to potential problems arising from lead poisoning.

## Case report

The patient was a 6-month-old female infant who had been exclusively breastfed. Neither of the parents was engaged in lead-related work. Since birth, the child lived in Longquanyi District, Chengdu, Sichuan Province, China. There was no known environmental lead pollution around her residential area. There was no previous medical history and no history of drug or food allergies. She underwent routine health care at the local public hospital on May 18, 2020. Her general examination was unremarkable, and her height and weight were normal. However, her blood lead level was more than 200.0 μg/L. The results of routine blood examination showed RBC = 5.44 × 10^12^/L, HGB = 115 g/L, MCV = 68.4 fl, MCH = 21.1 pg, and MCHC = 309 g/L. Her doctor diagnosed the patient with lead poisoning, and upon questioning, the mother denied that the infant had a history of exposure to any lead-containing substances such as medicine, food, paint and so on. Shengxuebao and zinc calcium gluconate oral liquid were given for 1 month. Following this, the patient's blood lead level was measured once again, and the result was 429.9 μg/L on June 4, 2020. After oral calcium supplementation for 1 month, the blood lead level remained elevated.

**Figure 1:**
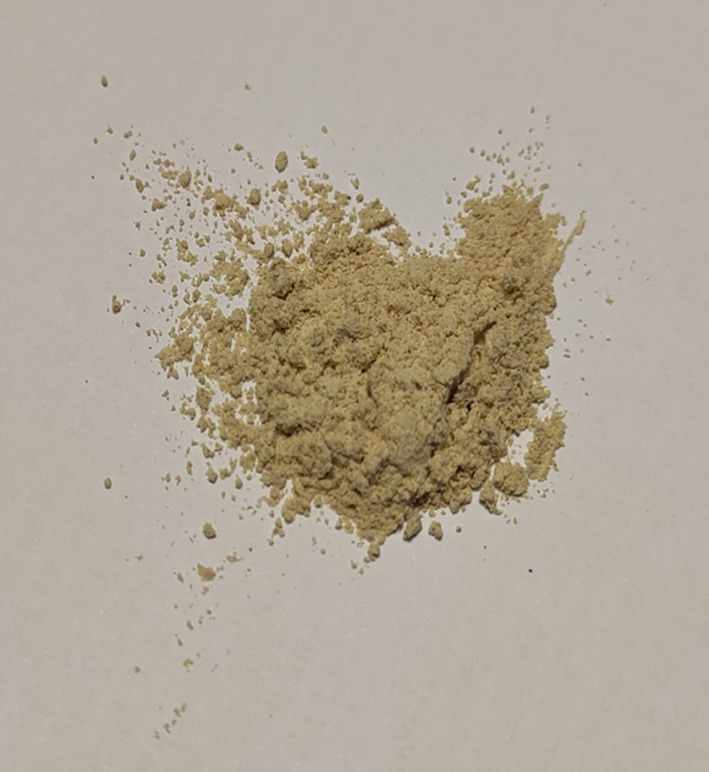
Samples of traditional Chinese medicine (TCM) were obtained from the patient.

The infant came to our hospital on June 28, 2020. The blood lead level and routine blood examination were re-evaluated. Her blood lead level was 454.0 μg/L. The results of routine blood examination showed RBC = 4.82 × 10^12^/L, HGB = 101 g/L, MCV = 63.9 fl, MCH = 20.9 pg, and MCHC = 327 g/L (microcytic hypochromic anemia). Because the infant was exclusively breastfed, we also checked the blood lead level of the mother and father. The results showed that the blood lead level of the mother was 77.0 μg/L and that of the father was 36.9 μg/L. The high blood lead level of the mother attracted our attention. After further probing the history of medication usage, we found that the mother had been using a kind of external traditional Chinese medicine—Hu Wang Fen (Figure 1). The lead content of the medicine was measured. The result was 180.1 ± 0.6 mg/kg, which exceeded the National Food Safety Standard (5 mg/kg) and the National Cosmetics Hygiene Standard (40 mg/kg). The mother was advised to stop using the medicine. In addition, we also tested the lead level of the mother's breast milk, and the result was 10.0 ± 0.7 μg/L, which was not significantly different from that of normal breast milk. Considering that the infant was too young for this topical medication, the pediatrician suggested that after stopping the exposure source, the infant should continue to take oral calcium supplementation to treat the elevated lead level and be reassessed 1 month later. The blood lead level was tested again on July 31, and the result was 435.0 μg/L.

**Figure 2:**
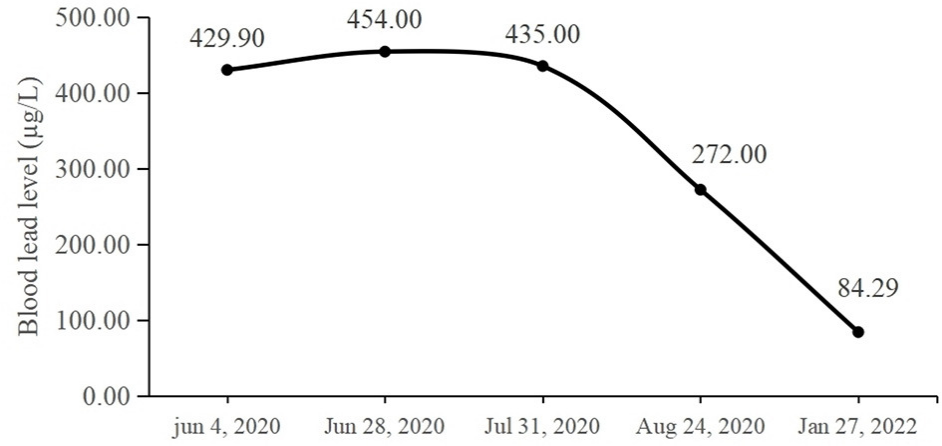
The blood lead levels of the patient were measured at different time points.

As the blood lead could not be reduced by oral calcium supplementation, the infant was admitted to the hospital. Chelation therapy was initiated, and edetate calcium disodium was administered at a dose of 200 mg by 12-h continuous intravenous infusion per day for 4 consecutive days for three cycles in total, with a 3-day rest period between cycles. 250 mg/d Calcium gluconate supplementation was also given every day. After three cycles of treatment, her blood lead level was tested again, and it was found that the level was reduced to 272 μg/L. The infant was discharged on August 24. She continued to take oral calcium supplementation after discharge. We also provided education to the mother about the common sources of lead exposure and the ways to prevent further lead exposure, for the health of both the infant and herself. Moreover, we made some suggestions on diet and nutrition, especially on calcium and iron intake. We re-tested the blood lead level after 5 months, and found that it was reduced to 84.29 μg/L on January 27, 2022. The changes in the patient's blood lead level are summarized in Figure 2.

## Discussion

Two documents entitled “The Children Lead Acidosis and Lead Poisoning Prevention Guide” and “Children's Blood Lead and Lead Poisoning Classification Principles” were issued by the Ministry of Health in China. It is emphasized that intervention is necessary when the blood lead level is more than 100 μg/L (6). The absorption rate of lead is high, and the excretion rate is low, which results in lead being more easily accumulated in children than in adults (7). The infant reported in this case had a hidden history of lead exposure and had no obvious symptoms. Infants and young children cannot express their own physical discomfort, and in addition to the more serious symptoms of toxic encephalopathy, general symptoms such as abdominal pain, irritability and memory loss are difficult to detect. The discovery of lead poisoning in this case was accidental, in that her child care program fortunately included a blood lead level test (there is no routine lead screening for children in China). Early screening of blood lead levels made this finding of lead poisoning as early as possible, thus avoiding the potential occurrence of serious complications and risk of damage to the nervous system.

The mother had applied Hu Wang Fen to her own armpit and the lower edge of her breast for a long time to keep these areas dry and refreshed. When the mother was asked whether the infant had a history of lead exposure, this medicine was not mentioned because it was not directly given to the infant. Child care doctors should endeavor to learn more about the possible sources of exposure to infant lead poisoning cases. In particular, they should be vigilant about the use of traditional medicines, and the parents' blood lead levels should be measured to further explore the source of lead intake. In this case, we were led to the discovery of the medicine that caused lead exposure because of the mother's high blood lead level, and subsequent test results confirmed that the topical medicine used by the mother contained lead. Due to hand-to-mouth and toy-to-mouth activities in children, the medicine may enter the human body through the digestive tract or skin-to-skin contact (8). The patient's blood lead level was much higher than her mother's, possibly because lead is more easily stored in children than in adults. Another reason is that lead is distributed differently in their bodies; 95% of lead is stored in bones in adults (9), while only 70% is stored in bones in children (10).

The antagonistic relationship between lead and calcium plays a critical role in the treatment of lead poisoning. Lead binds to the sites at which calcium typically acts and enters the cells through calcium channels. Therefore, calcium supplementation is beneficial to lead excretion (11). In the early stage, our patient was treated by oral calcium supplementation, but this proved ineffective. We postulate that the ineffectiveness of this treatment may have been for two reasons: first, the child remained exposed to the source of lead at the time, resulting in poor treatment effect, followed by anemia. The most important element in the treatment of lead toxicity is to disengage from the source as soon as possible. Second, the child was too young to have fully developed excretory functions, therefore the rate of effectiveness of the commonly used calcium supplement treatment was too slow. We no longer recommend this treatment to be used in patients of very young age who are being treated for high blood lead levels. For patients who are symptomatic or have a blood lead level of 450 μg/L or more, chelation treatment is generally recommended (12). Subsequently, when we added chelation therapy to the treatment program, the patient's blood lead level decreased rapidly. It should be noted that succimer is the first choice for chelation therapy in children since a weight of 8 kg and oral chelation therapy is safer. However, because only edetate calcium disodium was available at the hospital at the time of the patient's presentation, the doctor used edetate calcium disodium to remove lead.

According to the literature, the use of traditional medicine has become the main cause of lead poisoning in China. Ba-Baw san, Po-Ying-Tan, Jin Bu Huan, Hai Ge Fen, “Yi shao guang” ointment, and other traditional Chinese medicines all contain excessive lead levels (13). In Jiangxi, Fujian, Zhejiang, and Jiangsu provinces, Hong Dan Fen was used to prevent and treat diaper rash, infantile eczema, heat rash and other skin disorders. The children with lead poisoning caused by Huang Dan Fen mainly came from Shanghai and Hunan Province, where Huang Dan Fen was commonly used to prevent heat rash and symptoms of red buttocks in infants. The main components of these two lead-containing traditional Chinese medicines are lead trioxide and lead monoxide. On the basis of appearance and purpose, we speculated that the Hu Wang Fen used in this case was a type of Dan Fen.

Lead has no biologic role in the body, and any detectable level is abnormal. The deleterious effects of lead ingestion on children's neurocognitive and behavioral development are irreversible (8). Through awareness of this case, we appeal to women considering pregnancy and those who have young babies < 6 months of age to urge their medical providers to monitor children for blood lead levels for the early detection of lead poisoning. We suggest that in young children with high blood lead levels, the often used first line therapy of oral calcium supplementation should not be utilized as the sole therapy because the lead excretion capacity of young children is limited, and therefore the treatment benefit of calcium supplementation will be relatively slow. Infants, as well as their lactating mothers, should avoid using any unnecessary traditional Chinese medicine that may contain lead. Because blood lead testing is not a component of the routine child health care program, it is important for clinicians to have a more comprehensive understanding of the living habits, working environment, and drug history of children as well as that of their parents.

## Data availability statement

The original contributions presented in the study are included in the article/Supplementary material, further inquiries can be directed to the corresponding author.

## Ethics statement

Written informed consent was obtained from the patients legal guardian/next of kin for the publication of any potentially identifiable images or data included in this article. Written informed consent was obtained from the participant/patient(s) for the publication of this case report.

## Author contributions

All authors listed have made a substantial, direct, and intellectual contribution to the work and approved it for publication.
